# Mitral valve replacement for Libman–Sacks endocarditis in a patient with antiphospholipid syndrome secondary to systemic lupus erythematosus

**DOI:** 10.1093/jscr/rjy069

**Published:** 2018-04-03

**Authors:** Akio Nakasu, Tohru Ishimine, Hiroshi Yasumoto, Toshiho Tengan, Hidemitsu Mototake

**Affiliations:** Department of Cardiovascular Surgery, Okinawa Chubu Hospital, Okinawa, Japan

## Abstract

Libman–Sacks endocarditis is a relatively rare sterile verrucous vegetative lesion observed in systemic lupus erythematosus (SLE)/antiphospholipid syndrome (APLS) patients. Most patients with this condition are asymptomatic. Here we report a case of a 46-year-old woman with APLS secondary to SLE complicated with frequent thromboembolic events due to a mitral valve mass. We performed minimally invasive mitral valve replacement with a mechanical prosthetic valve, and she was successfully discharged 14 days after surgery. Thus, Libman–Sacks endocarditis may be an indication for mitral valve replacement.

## INTRODUCTION

Libman–Sacks endocarditis is a rare condition, which may be accompanied by antiphospholipid syndrome (APLS) secondary to systemic lupus erythematosus (SLE). Complications of Libman–Sacks endocarditis include thromboembolic events and valvular insufficiency and/or stenosis, and these are indications for surgery. Here, we present a case of mitral valve replacement with mechanical prosthesis in a 46-year-old woman with a history of APLS secondary to SLE with recurrent cerebral and myocardial infarctions.

## CASE REPORT

**Figure 1:**
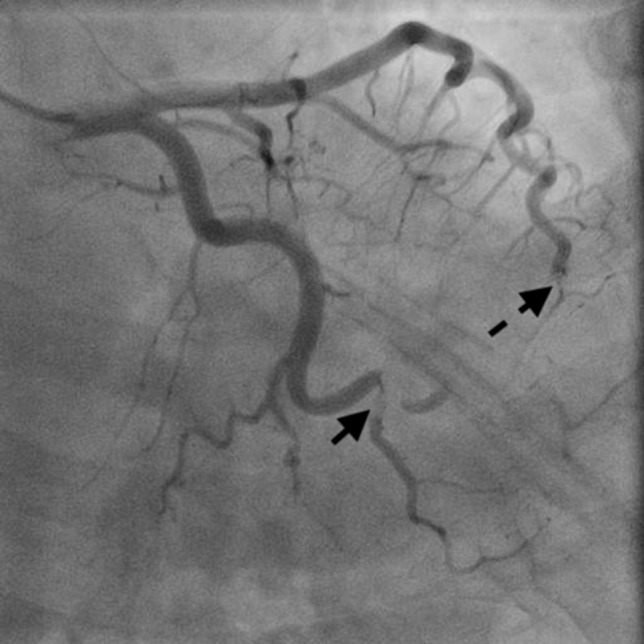
Coronary angiography showed obstructed distal left anterior descending artery (dotted arrow) and distal left circumflex artery (solid arrow).

**Figure 2:**
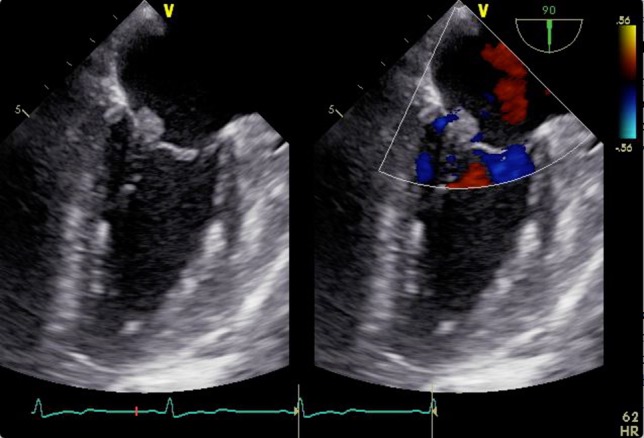
2D transesophageal echocardiogram showing a thickened mitral valve with vegetation that can be seen on the anterior mitral valve leaflet. Image on the right is a color flow Doppler view showing no mitral regurgitation and stenosis.

**Figure 3:**
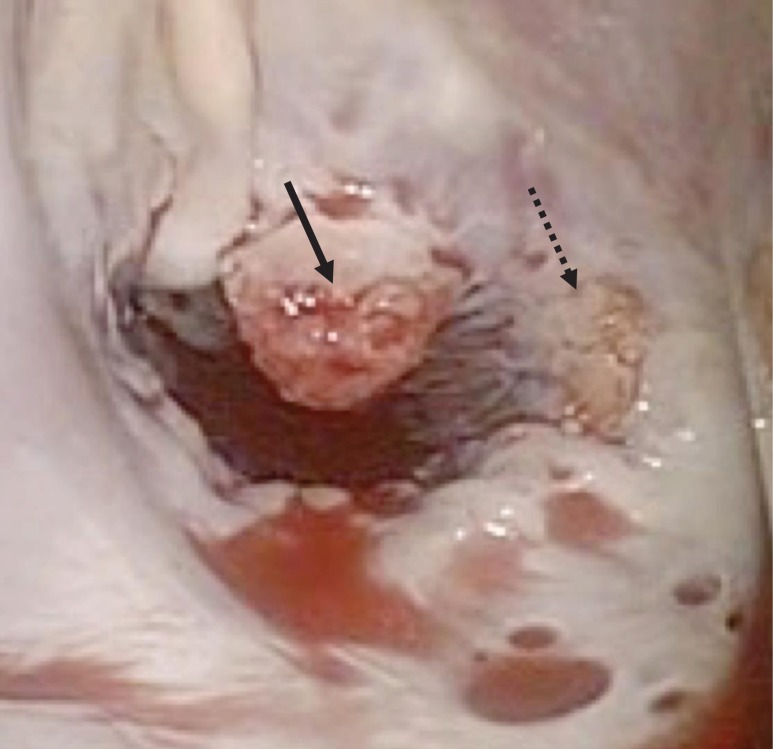
Intraoperative photograph showing verrucous vegetations on the mitral valve of A2 (solid arrow) and A3 (dotted arrow).

A 46-year-old woman with a history of long-standing SLE and APLS for >17 years and two recent cerebral infarctions was referred to our emergency department with a sudden-onset chest pain and dyspnea (New York Heart Association functional class III). Her usual steroid dose was 10 mg per day. Secondary to SLE associated thrombocytopenia she was anticoagulated with dabigatran. The EKG was consisted with anterior wall STEMI. Troponin level was 1.850. Coronary angiography showed obstruction at the distal left anterior descending and left circumflex arteries (Fig. 1). Transesophageal echocardiography demonstrated a preserved ejection fraction of the left ventricle and a mobile vegetation measuring 9 × 6 mm^2^ at the mitral valve (Fig. 2). Blood cultures were all negative. She urgently underwent mitral valve replacement through mini right thoracotomy to prevent further thromboembolic events. Cardiopulmonary bypass was undertaken femoral arterial and femoral and internal jugular venous cannulation. We did not perform coronary artery bypass graft because the obstructed arteries were quite distal. We identified a mass at the mitral A2 segment rough zone and two very small lesions at the A3 segment without annular calcification or submitral apparatus disease (Fig. 3). A2 segment was largely affected and shortened; therefore, we decided to not proceed the valve repair. Replacement was performed with a 27/29 mm On-X mechanical prosthesis (Medical Carbon Research Institute, LLC, Austin, TX, USA). Anterior and posterior chordae were spared. Aortic cross clamp time was 242 min and cardiopulmonary bypass time was 289 min. A pathological examination of excised valve leaflets demonstrated a fibrin thrombus without an evidence of microorganisms. The postoperative course was uneventful, and she was discharged 14 days after the surgery. She was followed up for 6 months, and her condition was stable.

## DISCUSSION

According to the report, the prevalence of cardiovascular disease in patients with SLE/APLS has been estimated to be >50% [1, 2]. Approximately 50–60% of patients with SLE and APLS reveal a disease of one or more valves being frequently observed in the left side [3]. Indication for surgery is usually heart failure due to the insufficiency of mitral or aortic valve. Mitral stenosis is the least (<5%) prevalent valve disease and is often associated with mitral regurgitation [1]. This case is relatively rare in that the indication of surgery was frequent thromboembolic events.

In general, mitral valve repair is the gold standard surgery for mitral regurgitation; however, it is interesting that mitral valve repair has been tried in only 15% of patients with secondary APLS [3]. The reason of this tendency is unclear; however, we believe secondary APLS patients may be predisposed to multiple and large vegetations and the valve was injured with fibrosis or thickening.

In terms of the valve replacement, prosthetic valve selection between mechanical and bioprosthetic valve is till controversial. The rather young age of the patients for this disease seem to make a mechanical valve as a first choice. However, thromboembolic events of mechanical valve may lead to fatal outcomes and the maintenance of much higher level of INR between 3 and 3.5 also may contribute to bleeding complications. One of the advantage of bioprosthetic valve is the independence from anticoagulation monitoring but as Sladek and Accola [4] mentioned, long-term durability under hypercoagulative status is unclear. Even though which valve was selected, it is important that we should follow up the patient diligently for the better outcome.

Mortality after surgery is relatively high, and most of these are myocardial and cerebral complications and other overt thromboembolic events [3]. It is important to maintain INR at much higher levels than normal (3–3.5) to prevent thrombus formation [5].
